# Missed Opportunities along the Prevention of Mother-to-Child Transmission Services Cascade in South Africa: Uptake, Determinants, and Attributable Risk (the SAPMTCTE)

**DOI:** 10.1371/journal.pone.0132425

**Published:** 2015-07-06

**Authors:** Selamawit Woldesenbet, Debra Jackson, Carl Lombard, Thu-Ha Dinh, Adrian Puren, Gayle Sherman, Vundli Ramokolo, Tanya Doherty, Mary Mogashoa, Sanjana Bhardwaj, Mickey Chopra, Nathan Shaffer, Yogan Pillay, Ameena Goga, South African PMTCT Evaluation (SAPMCTE) Team

**Affiliations:** 1 Health Systems Research Unit, Medical Research Council, Cape Town, South Africa; 2 School of Public Health, University of the Western Cape, Cape Town, South Africa; 3 United Nations Children’s Fund, New York, United States of America; 4 Centers for Disease Control and Prevention, Center for Global Health, Division of Global HIV/AIDS, Atlanta, Georgia, United States of America; 5 National Institute for Communicable Diseases of the National Health Laboratory Services, Johannesburg, South Africa; 6 Division of Virology and Communicable Diseases, Faculty of Health Science, University of the Witwatersrand, Johannesburg, South Africa; 7 Department of Paediatrics and Child Health, Faculty of Health Sciences, University of the Witwatersrand, Johannesburg, South Africa; 8 School of Public Health, University of the Witwatersrand, Johannesburg, South Africa; 9 Centers for Disease Control and Prevention, Center for Global Health, Division of Global HIV/AIDS, Pretoria, South Africa; 10 United Nations Children’s Fund, Pretoria, South Africa; 11 World Health Organization, Geneva, Switzerland; 12 National Department of Health, Pretoria, South Africa; 13 Department of Paediatrics and Child Health, University of Pretoria, Pretoria, South Africa; 14 Health Systems Research Unit, Medical Research Council, Pretoria, South Africa; Tulane University School of Public Health, UNITED STATES

## Abstract

**Objectives:**

We examined uptake of prevention of mother-to-child HIV transmission (PMTCT) services, predictors of missed opportunities, and infant HIV transmission attributable to missed opportunities along the PMTCT cascade across South Africa.

**Methods:**

A cross-sectional survey was conducted among 4–8 week old infants receiving first immunisations in 580 nationally representative public health facilities in 2010. This included maternal interviews and testing infants’ dried blood spots for HIV. A weighted analysis was performed to assess uptake of antenatal and perinatal PMTCT services along the PMTCT cascade (namely: maternal HIV testing, CD4 count test/result, and receiving maternal and infant antiretroviral treatment) and predictors of dropout. The population attributable fraction associated with dropouts at each service point are estimated.

**Results:**

Of 9,803 mothers included, 31.7% were HIV-positive as identified by reactive infant antibody tests. Of these 80.4% received some form of maternal and infant antiretroviral treatment. More than a third (34.9%) of mothers dropped out from one or more steps in the PMTCT service cascade. In a multivariable analysis, the following characteristics were associated with increased dropout from the PMTCT cascade: adolescent (<20 years) mothers, low socioeconomic score, low education level, primiparous mothers, delayed first antenatal visit, homebirth, and non-disclosure of HIV status. Adolescent mothers were twice (adjusted odds ratio: 2.2, 95% confidence interval: 1.5–3.3) as likely to be unaware of their HIV-positive status and had a significantly higher rate (85.2%) of unplanned pregnancies compared to adults aged ≥20 years (55.5%, p = 0.0001). A third (33.8%) of infant HIV infections were attributable to dropout in one or more steps in the cascade.

**Conclusion:**

A third of transmissions attributable to missed opportunities of PMTCT services can be prevented by optimizing the uptake of PMTCT services. Identified risk factors for low PMTCT service uptake should be addressed through health facility and community-level interventions, including raising awareness, promoting women education, adolescent focused interventions, and strengthening linkages/referral-system between communities and health facilities.

## Introduction

Eliminating mother-to-child HIV transmission (MTCT) is now considered a realistic public health goal for resource limited settings.[1] Clinical trials have shown that providing the most effective antiretroviral (ARV) regimen for HIV-positive pregnant women can reduce the risk of MTCT to less than 2% in non-breastfeeding populations and to <5% in breastfeeding populations.[2–4]

The prevention of mother-to-child HIV transmission (PMTCT) ARV coverage for Sub-Saharan African countries in 2012 is reported at 65%, far short of the 90% global target.[5] South Africa has made rapid progress and is one of those countries that are close to achieving the goal of providing ARV regimens to 90% of pregnant women living with HIV. In 2012 the PMTCT service coverage for South Africa was reported at 83%.[5] Reports from two consecutive facility-based national surveys also show a declining trend in early (4–8 weeks) transmission rates (2010: 3.5%, 95% confidence interval (CI): 2.9–4.1%; 2011: 2.7%, 95%CI:2.9–4.1%) from transmission rates reported in 2008 (7.1%, 95% CI: 6.2–8.0%).[6–9] However, despite the overall progress, there is considerable variation in coverage of the cascade of PMTCT services across South Africa: reported attrition rates range from 5.3% to 20.6% at antenatal HIV testing service points, and 22% to 50% at ARV initiation.[10–15] These findings suggest that MTCT rates could be further lowered through reducing differential uptake of PMTCT services.

A detailed analysis of the PMTCT continuum of care (often referred to as the “PMTCT cascade”) tailored to the local context could assist in identifying and prioritizing factors that can be targeted for intervention. Population attributable fraction (PAF) is a useful indicator for demonstrating the relative importance of modifiable risk factors, essentially providing estimates of how much of a disease could potentially be eliminated if risks identified are mitigated and/or eliminated from the population.

In the above context, this study aimed to address the following three objectives in the South Africa programme:

(1) To measure national uptake of antenatal and early postnatal PMTCT services; (2) to identify key dropout points, dropout rates and determinants of dropouts in the PMTCT cascade; and (3) to estimate the PAF of infant HIV infection associated with dropout in the PMTCT continuum of care.

## Methods

### Study design

A cross-sectional survey was conducted from June-December 2010 among mother/caregiver-infant pairs visiting the immunisation service points of randomly selected public primary health care facilities (PHCs) and community health centres (CHCs) across the nine provinces of South Africa. The protocol and overall transmission rate findings have been published elsewhere.[8]

At the time of the study guidelines in South Africa recommended that mothers be offered Zidovudine (AZT) from 28 weeks gestation and single dose nevirapine (sdNVP) at labour or triple (combination) antiretroviral therapy (cART) if CD4 cell count was ≤200 cells/mm3. Infants were offered sdNVP at birth and 7–28 days of postpartum AZT.[16]

### Sample size and Sampling

The sample size was calculated taking into account the 2009 antenatal HIV prevalence data[17], transmission rate estimates from two previous sub-national surveys[18, 19], and the coverage of ARV prophylaxis in each province from district health information system (DHIS) reports. A precision-based sample size was calculated for varying MTCT precision levels by province (ranging from 1% to 2%) and a design effect of 2 to account for clustering within health facilities. Based on this, the collection of interview data and infant dried blood spots (DBS) from 12,200 mother-infant pairs was needed to provide stable provincial and national level estimates of transmission rates and PMTCT services uptake. Further details on parameters used for sample size calculation are presented elsewhere.[6]

The sampling frame comprised all public PHCs and CHCs throughout the country. Small facilities (with <130 annual Diptheria-Pertussis-Tetatus-1 (DTP1) immunisations) were excluded from the sampling frame [the proportion of annual six-week immunisation done in small facilities in 2007 was only 6.7%]. A multistage stratified cluster sampling method was used to select facilities. The 2007 DHIS immunisation data and the 2009 antenatal HIV prevalence estimates were used to stratify facilities into three groups: medium-sized facilities with 130–300 annual DTP1 immunisation number, large (≥ 300 annual DTP 1 immunisation number) facilities with HIV prevalence below the national HIV prevalence estimate (<29%), and large facilities with HIV prevalence ≥29%. A probability proportional to size sampling method was used to select 580 medium and large size facilities with both high and low HIV prevalence. At the final step, a fixed number (proportional to facility size) of individual mother-infant pairs were sampled consecutively from each selected facility within a specified period of time [3 (in 8 provinces) to 4 (in 1 province) weeks was spent in each facility].

### Data collection and Laboratory procedures

Mother/caregiver-infant pairs at selected facilities were approached and screened for eligibility. Inclusion criteria were infants aged 4-8weeks, with no emergency illness and receiving their six-week immunisation on the visit day. Consented mothers were interviewed on antenatal and obstetric history, knowledge about PMTCT, history of antenatal HIV testing, HIV status, CD4 count testing and ARV services received. PMTCT-related questions were answered by biological mothers only. Interview data were collected on hand-held devices (cell phone pre-programmed questionnaire).

After the interview, individual pre-test counselling was given to each mother and if mothers consented, DBS were collected from infants using heel-prick. DBS specimens were tested for HIV antibodies by means of an enzyme immunoassay (EIA) (Genscreen HIV1/2 Ab EIA Version 2, Bio-Rad Laboratories, France). A positive EIA result indicated infant HIV exposure. A qualitative HIV polymerase chain reaction (PCR) (COBAS AmpliPrep/COBAS TaqMan (CAP/CTM) Qualitative assay version 1.0 assay, Roche Diagnostics, Branchburg, NJ) test was performed on all EIA-positive DBS to determine whether the infant was HIV PCR-positive (i.e. HIV-infected).

### Statistical analysis

Infants were included in this analysis if they had both maternal interviews on the PMTCT sections of the questionnaire and complete laboratory results for HIV testing (i.e. EIA test result was needed from all infants and for EIA-positive infants PCR test result was needed in order to be included in the analysis). Mothers who had an EIA-positive infant were considered to be HIV-positive. We identified four PMTCT cascade steps (also referred as ‘PMTCT cascade indicators’): maternal HIV-positive status knowledge (reporting HIV-positive status determined before or during pregnancy), maternal CD4 cell count testing and receipt of result, receipt of maternal ARV and infant nevirapine (NVP). The denominator for each of the 4 indicators was “total number of HIV-positive mothers, as identified from infant EIA test”. Dropout was calculated as a proportion of HIV-positive mothers who missed a step in the cascade regardless of returning to receive subsequent services. Mothers who miss multiple steps are considered as dropout only once, at their first missed step. Dropout does not take poor adherence into consideration; rather it only included instances where mother-infant pairs did not access a given service.

We compared categorical responses using chi-square tests. Multivariable regression analysis was used to identify factors associated with missed opportunities and dropouts in the following PMTCT cascade indicators: (1) maternal HIV-positive status knowledge (2) CD4 cell count testing and receipt of result (as a single outcome of interest), and (3) an overall indicator for “at least one dropout at any of the four PMTCT cascade steps”. Initial univariable analysis included explanatory variables identified in similar studies and those with biological plausibility. All variables with a p-value below 0.2 were included in the starting variable set for multivariable regression. From a multivariable model, factors were dropped if they were non-significant (at p 0.05) and did not markedly alter (by 10% or more) estimates of other significant variables in the model. We created a socio-economic score from availability of assets (television, car, refrigerator, stove), and dwelling characteristics (type of water, toilet, fuel and building material) using the principal component analysis method.

### Population attributable fraction (PAF)

In order to assess the potential for prevention, this study used the method explained by Basu and Landis to estimate PAF.[20] Two types of PAF were estimated. The first PAF was estimated assuming that prevention efforts/interventions target each cascade step separately; the estimated PAF from this step reflects the transmission preventable by eliminating missed opportunities from one cascade step. The second PAF—which we refer as cumulative PAF- was estimated assuming prevention interventions target 2, 3 or all 4 cascade steps at once. We report incidence reductions that correspond to the cumulative PAF.

To estimate both the PAF and the cumulative PAF, 8 models (1 PAF model and 1 cumulative PAF model for each of the 4 cascade steps) were fitted at each of the four PMTCT cascade indicators and the outcome ‘infant HIV infection’. Each model was adjusted for potential covariates from the literature, which included maternal age (10 year age groups), education (below secondary), socioeconomic score (the two lowest quintiles, middle and fourth quintile, vs. fifth/highest quintile), feeding pattern (exclusive breastfeeding or no breastfeeding vs. mixed breastfeeding), number of children (1, 2, ≥3), and type of delivery (caesarean section or vaginal delivery). Intermediary factors that are on the causal pathway between the PMTCT cascade indicators and HIV transmission risk were not included in the analysis. Covariates with missing data used for adjusting the PAF models were imputed sequentially using Monte Carlo multiple imputation technique. Cascade indicators with missing data were not imputed as the missing responses of these indicators are correlated. We had 4.7%, 1.4% and 2.2% missing responses for CD4 count testing, and maternal and infant ARV initiation indicators, respectively. The PAF estimates were analysed using all available data. The cumulative PAF was estimated using complete case analysis by excluding observations with missing cascade indicator data. Analyses were done using STATA SE (version 12, Texas, 77845 USA) and were adjusted for the complex survey design, including sample size realization, clustering and the 2010 live-birth distribution across provinces.

The study protocol was approved by the United States Centers for Disease Control and Prevention and the institutional review board of the Medical Research Council of South Africa. All study participants provided written informed consent.

## Results

**Fig 1:**
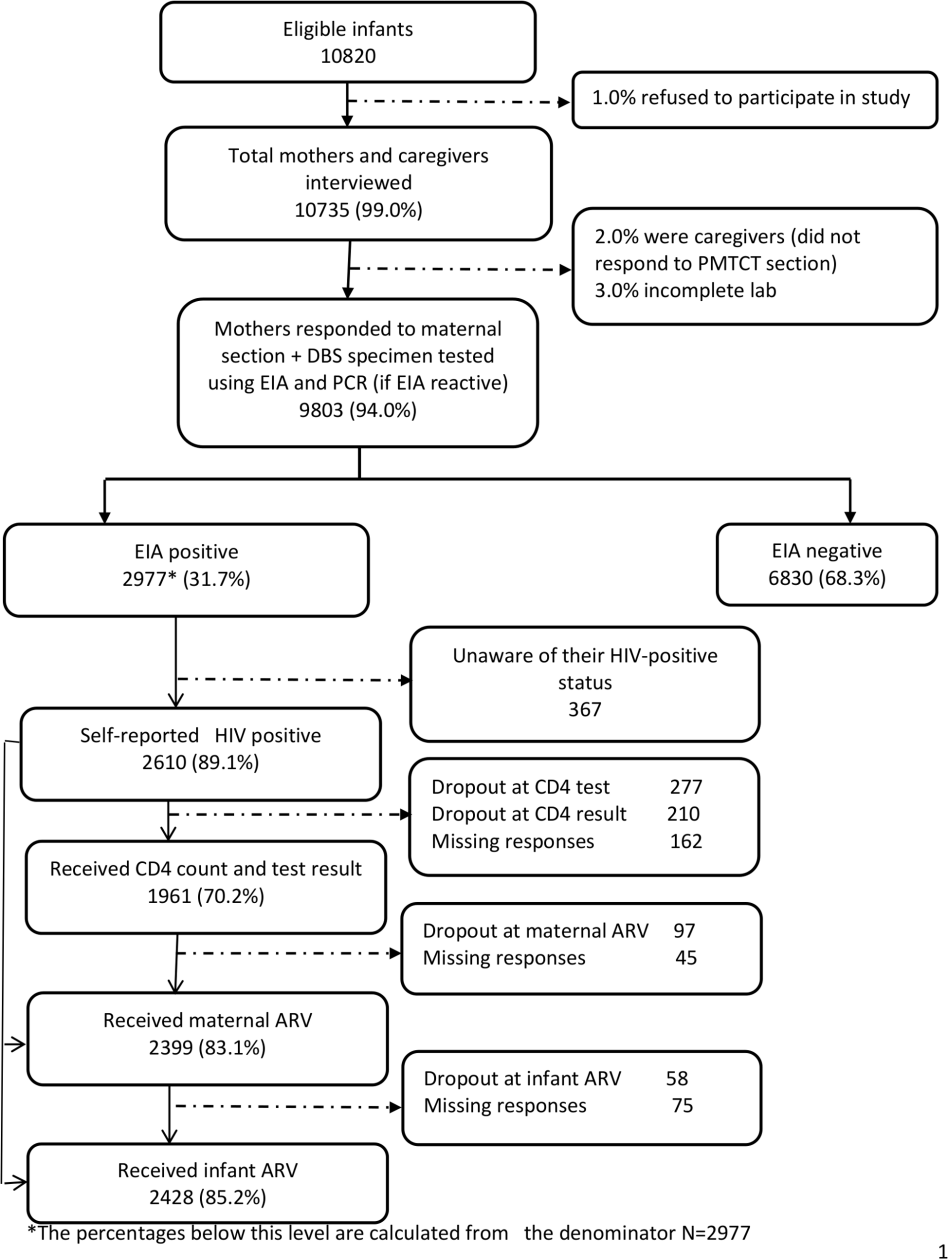
2010 PMTCT cascade Study Profile.

A total of 10,820 mothers/caregivers who came to selected health facilities for their infants’ six-week immunisation service were approached and screened for study eligibility (Fig 1). Ninety-nine percent (10,735) of mother/caregivers screened were eligible and consented to be interviewed; of these, 9,803 (94%) who responded to maternal sections of the questionnaire and had complete infant laboratory results were included in this analysis

The majority of participants were single mothers (74.3%), aged less than 30 years (inter-quartile range 21–30), of African/Black race (92.9%), and with some high school education (77.7%). Most (60.1%) pregnancies were unplanned. Most mothers attended their first antenatal care visit in their second (57.9%) or third (15.4%) trimester. Forty percent (40.2%) of mothers were primipara (Table 1). Timing of first antenatal care visit did not differ by maternal age group (category: <20 years vs. ≥20 years) or number of children. However, adolescent mothers (age <20 years) had a higher unplanned pregnancy rate (85.2%) than adult (age ≥20 years) mothers (55.5%, p = 0.0001).

**Table 1:** Characteristics of mother-infant pairs in the 2010 PMTCT cascade study

Maternal and Infant Characteristics	Weighted N = 1171637	
	Weighted %	Weighted 95% CI
**Maternal Age, years**		
<15	0.09	4.7–19.3
15–19	15.5	14.5–16.5
20–24	30.6	29.6–31.7
25–29	26.1	25.1–27.2
30–34	16.7	15.8–17.6
35–39	8.4	7.7–9.1
40–44	2.4	2.1–2.8
≥45	0.2	0.1–0.4
**Maternal Age**		
Median (Inter quartile range)	25 (21–30)	
Mean (standard deviation)	26 (6.2)	
**Maternal highest school grade passed**		
None	2.0	1.7–2.4
Grade 1–7	15.1	13.9–16.3
Grade 8–12	77.7	76.2–79.1
Above Grade 12	5.2	4.5–6.1
**Marital status**		
Single	74.3	72.3–76.2
Married/cohabiting	25.3	23.4–27.3
Windowed/Divorced	0.4	0.3–0.6
**Socio-economic score** *		
Poorest 20%	20.4	18.3–22.6
Second	19.9	18.6–21.4
Third	21.2	19.8–22.7
Fourth	29.1	27.0–31.3
Highest 20%	9.5	8.3–10.7
**No of live children**		
1	40.2	38.9–41.6
2	32.2	31.0–33.4
≥3	27.6	26.4–28.9
**Planned Pregnancy**		
Yes	39.9	38.0–41.8
No	60.1	58.2–62.0
**Reported Gestational age at first ANC**		
1^st^ trimester	26.7	25.2–28.2
2^nd^ trimester	57.9	56.3–59.5
3^rd^ trimester	15.4	14.1–16.8
**Infant gender**		
Male	50.6	49.4–51.8
Female	49.4	48.2–50.6
**Infant Race**		
Black	92.9	91.2–94.3
Coloured	6.0	4.7–7.6
White, Indian and other	1.1	0.8–1.6

Category frequencies do not add to the total because of missing responses; ANC–antenatal care

*the socio-economic score was constructed from the following assets (television, car, refrigerator, stove), and dwelling characteristics (water, toilet, fuel and building material). Participants in the 4th level had most basic assets/utilities in the house (including flush toilet, pipe water (in the house), stove, refrigerator, TV, electricity, gas or paraffin for cooking and brick/cement house). The participants in the 3rd level and below categories do not have at least one of the assets or utilities that are mentioned for level 4. Participants in Category 5 had car in addition to the assets/utilities mentioned for level 4.

Of the 9,803 mothers interviewed, 98.8% (9,679) reported they had been tested for HIV during an antenatal visit (with their recent pregnancy) or were diagnosed with HIV infection prior to pregnancy. Almost all (97.8%) mothers who were HIV tested received their test results. Amongst mothers who reported receiving their HIV test result, 29.1% (2,610) reported being HIV-positive compared to 31.7% (2,977) who were positive by infant EIA tests (this difference was not statistically significant).

Of the 2,977 HIV-positive mothers (based on the infant EIA test), 89.0% (2,610) self-reported HIV-positive status, 8.6% reported HIV-negative status and 2.4% reported unknown HIV status—thus, 11.0% (367) of HIV-positive mothers were reportedly unaware of their HIV-positive status (Fig 1 and Table 2).

**Table 2:** Percentage of infant HIV transmission (at 4–8 weeks) attributable to dropouts along the PMTCT cascade, national survey, South Africa

Dropout at key antenatal and early postnatal PMTCT steps	Uptake and dropout		Population Attributable Fraction (PAF)*	Cumulative PAF*+	Infant HIV incidence in observed data*	Infant HIV incidence if observed dropout is eliminated* Ⱶ
	Unweighted n	Weighted %	% (95% CI)	% (95% CI)	% (95% CI)	% (95% CI)
	N = 2977		N = 2977	N = 2768	N = 2768	N = 2768
**Maternal HIV status knowledge**						
Unaware of their HIV positive status	367	11.0	15.3 (5.6;24.0)	16.7 (6.0;26.1)	3.4 (2.8;4.1)	2.8 (2.2;3.6)
Know they are positive	2610	89.0				
**CD4 count testing and result**						
Dropout at CD4 test	277	9.7				
Dropout at CD4 result	210	8.6		23.2 (5.0;37.9)	2.8 (2.2;3.6)	2.6 (1.9;3.5)
Total not receiving this step	854	29.8	23.2 (5.3;37.8)			
Retained	1961	70.2				
Maternal cART/AZT**						
Dropouts at this step	97	3.2		29.1 (10.0;44.1)	2.6 (1.9;3.5)	2.4 (1.7; 3.3)
Total not receiving this step	533	16.9	26.6 (13.0;38.1)			
Retained	2399	83.1				
**Infant Nevirapine** **						
Dropouts at this step	58	1.7		33.8 (14.3;48.8)	2.4 (1.7; 3.3)	2.2 (1.6;3.1)
Total not receiving this step	474	14.8	19.4 (7.8;29.5)			
Retained	2428	85.2				
**All 4 services**						
At least 1 missed step	1009	34.9		33.8 (14.3;48.8)†	3.4 (2.8;4.1)	2.2 (1.6;3.1)
Completed all steps	1786	65.1				

PMTCT–Prevention of Mother-to-child Transmission; ARV–Antiretrovirals; cART–triple antiretroviral therapy; AZT–Zidovudine; CI: confidence interval. N–number (overall frequency) n—number (category frequency).

‘Total not receiving this step’ includes those who are not receiving the given step because of missed opportunities in prior cascade steps (e.g. for CD4 count ‘total not receiving is calculated as 367 (who missed both CD4 and HIV status knowledge) +210+277 who missed CD4 count test or result.)

‘Dropouts at this step’ refers to new dropouts occurred at each step. Mothers who dropout at two or more consecutive steps are counted as dropout only once at their first dropout point e.g. for ‘CD4 count test’ new dropouts are 277 (this does not include the 367 who missed both CD4 and HIV status knowledge)

The PAF was estimated for each ‘Total not receiving this step’ using all available information. Cumulative PAF was estimated for each ‘Dropout at this step’ using observations with complete data for all 4 uptake indicators (N = 2768).

Column2:

Each percentage for uptake, dropout and ‘total not receiving this step’ are calculated from the denominator N = 2977.

We had 162 (4.7%) missing response for CD4 count question; 45 (1.4%) missing response for maternal ARV question; 75 (2.2%) missing response for infant Nevirapine question.

Due to the above missing responses, the following do not add-up up to the total N / %: percent dropout from each step do not add to the total ‘% at least missed 1 service’; category frequencies do not add to the total ‘N’

In total 8 models were fitted to estimate the PAF and cumulative PAF for the 4 cascade steps. Each of the models were adjusted for maternal age (<20 years, 20–29 years, 30–39 years, >40 years), education (below secondary), socioeconomic score (the 1st two quintiles, middle and fourth quintile vs. last quintile), feeding pattern (exclusive breastfeeding or no breastfeeding vs. mixed breastfeeding), number of live children (1, 2, ≥3), and type of delivery (caesarean section vs. vaginal delivery).

*adjusted estimates

**dropout refers to non-receipt of ARVs and does not examine adherence.

^╫^ The cumulative PAF for at least 1 missed step comprises the 16.7% PAF at the maternal HIV status knowledge stage and increases by 6.5% at the CD4count stage; and then by an additional 5.9% and 4.7% at maternal and infant ARV steps respectively, giving a total cumulative PAF (for ‘at least 1 missed step’) of 33.8%

^├^ The biggest reduction (by 0.6% from 3.4% to 2.8%) in infant HIV incidence occurs when missed opportunities in maternal HIV status knowledge is eliminated

In multivariable analysis, mothers were more likely to be unaware of their HIV-positive status if they had a home delivery (adjusted odds ratio—AOR: 1.9, 95% CI: 1.2–3.0), maternal age <20 years (AOR: 2.2, 95%CI:1.5–3.3), late presentation (during the 3^rd^ trimester) for antenatal care (AOR: 1.7, 95%CI:1.1–2.6), or were primiparous (AOR: 1.8, 95%CI:1.3–2.5) (Table 3).

**Table 3:** Factors associated with dropout at the stage of maternal HIV testing (A), CD4 count testing and result (B) and at any of the four steps in the PMTCT cascade(C), 2010 (logistic model).

	N	(A) Missed opportunities due to unawareness of HIV-positive status (un-weighted N = 2977)		(B) Dropout at CD4 count testing or not receiving result (un-weighted N = 2448)		(C) Dropout in at least one of the CD4, maternal or infant ARV services or being unaware of HIV-positive status (un-weighted N = 2795)	
		Number (%)	AOR (95%CI)	Number (%)	AOR (95%CI)	Number (%)	AOR (95%CI)
**Home delivery**							
Yes	193	43 (18.7)	1.9 (1.2–3.0)	44 (31.7)	1.4 (0.9–2.1)	107 (55.4)	2.1 (1.5,2.9)
No	2784	324 (10.5)	1.0	443 (20.0)	1.0	902 (33.7)	1.0
**Timing of first ANC visit**							
1^st^ trimester	671	73 (9.4)	1.0	89 (15.6)	1.0	193 (29.2)	1.0
2^nd^ trimester	1438	166 (9.7)	1.0 (0.7–1.4)	226 (20.3)	1.4 (0.9–1.9)	460 (33.2)	1.2 (0.9,1.5)
3^rd^ trimester	352	52 (14.0)	1.7 (1.1–2.6)	70 (24.3)	1.6 (1.1–2.5)	149 (42.1)	1.7 (1.3,2.4)
Missing	516	76 (14.3)	1.4 (0.9–2.1)	102 (25.2)	1.6 (1.1–2.4)	207 (41.8)	1.5 (1.1,2.1)
**Socioeconomic quintiles**							
Poorest 20% and Second poorest	1317	179 (12.7)	1.2 (0.9–1.7)	267 (17.5)	1.3 (1.1–1.7)	515 (40.4)	1.3 (1.1–1.6)
Third, Fourth and Highest 20%	1658	188 (9.6)	1.0	220 (24.8)	1.0	494 (30.8)	1.0
**Maternal Age**							
<20 years	185	52 (24.7)	2.2 (1.5–3.3)	37 (30.0)	1.7 (1.1–2.7)	99 (53.2)	2.0 (1.4,2.8)
20–39 years	2699	300 (9.9)	1.0	432 (19.9)	1.0	870 (33.5)	1.0
≥ 40 years	66	11 (15.8)	1.8 (0.8–3.8)	14 (31.2)	1.3 (0.6–2.9)	32 (51.8)	1.8 (0.9–3.5)
**Number of live children**							
1	842	142 (15.4)	1.8 (1.3–2.5)	129 (20.3)	1.0 (0.7–1.4)	310 (37.6)	1.2 (0.9–1.6)
2	1134	118 (9.3)	1.1 (0.8–1.6)	184 (20.1)	1.1 (0.8–1.4)	356 (33.0)	1.1 (0.9,1.3)
>3	1001	107 (9.4)	1.0	174 (21.6)	1.0	343 (35.3)	1.0
**Education**							
Below Secondary or No schooling	664	83 (12.0)	1.0 (0.7–1.4)	149 (29.3)	1.6 (1.2–2.1)	269 (44.1)	1.4 (1.1,1.8)
Secondary or above	2311	283 (10.7)	1.0	338 (18.5)	1.0	739 (32.8)	1.0
**Disclosed to at least one person**							
Yes	2118			390 (19.4)	1.0		
No	330			97 (29.2)	1.6 (1.2–2.3)		

Percentages are row percentages. The following factors were non-influential in a univariable analysis (for all models) thus were not included in the multivariable model: lack of social Support, inadequate knowledge of mother-to-child transmission modes, unplanned pregnancy, married or living with partner, and race. Disclosure of status was asked only if the mother know her HIV status. All numbers (N, n) are unweighted and all percentages and AORs’ are from weighted analysis. Category frequencies do not add to the total because of missing responses.

The significance (p.value) of the overall effects for the following multiple category variables were: for first antenatal visit 0.04, 0.049 and 0.005 for models A- C respectively; for number of live children 0.0006, 0.9,0.3 for models A-C respectively and for maternal age 0.0002, 0.04, 0.002 for models A-C respectively.

TBAs–traditional birth attendant; ANC—antenatal; ARV- antiretroviral

Of the 2,977 HIV-positive mothers (based on the infant EIA test), 78.8% (2,171) had a CD4 count test and 70.2% (1,961) received their CD4 count test result; 9.7% did not have a CD4 count test, and 8.6% did not receive their CD4 count test result (Table 2). In a multivariable analysis (Table 3), dropout at CD4 count test/result was significantly associated with non-disclosure of HIV status (AOR: 1.6, 95%CI: 1.2–2.3), maternal age <20 years (AOR: 1.7, 95%CI: 1.1–2.7), delayed (during the 3^rd^ trimester) first antenatal visit (AOR: 1.6, 95%CI: 1.1–2.5), educational status below secondary level (AOR: 1.6, 95%CI: 1.2–2.1) and low (the first two lowest quintiles) socioeconomic scores (AOR: 1.3, 95%CI: 1.1–1.7).

Of all HIV-positive mothers, 83.1% (2399) received some maternal ARV (30.5% cART and 52.6% AZT), and 85.2% (2428) of their infants received NVP (Table 2). Overall, 80.4% (2276) of HIV-positive mothers and their infants received both maternal (AZT and cART) and infant (NVP or AZT) ARV. The dropout rate at maternal and infant ARV steps was 3.2% and 1.7%, respectively (Table 2).

Overall, 34.9% of mothers missed at least one step in the cascade, whilst 65.1% of mothers successfully received all 4 (HIV testing, CD4 count test/result, maternal and infant ARV) key antenatal and peri-partum PMTCT services (Table 2). Most factors identified as influential on separate models fitted for HIV status knowledge and CD4 count testing/result were also influential on the combined indicator “dropout at any one point in the cascade” (Table 3).

### Dropout rates, population attributable fraction (PAF), and transmission rates

**Fig 2:**
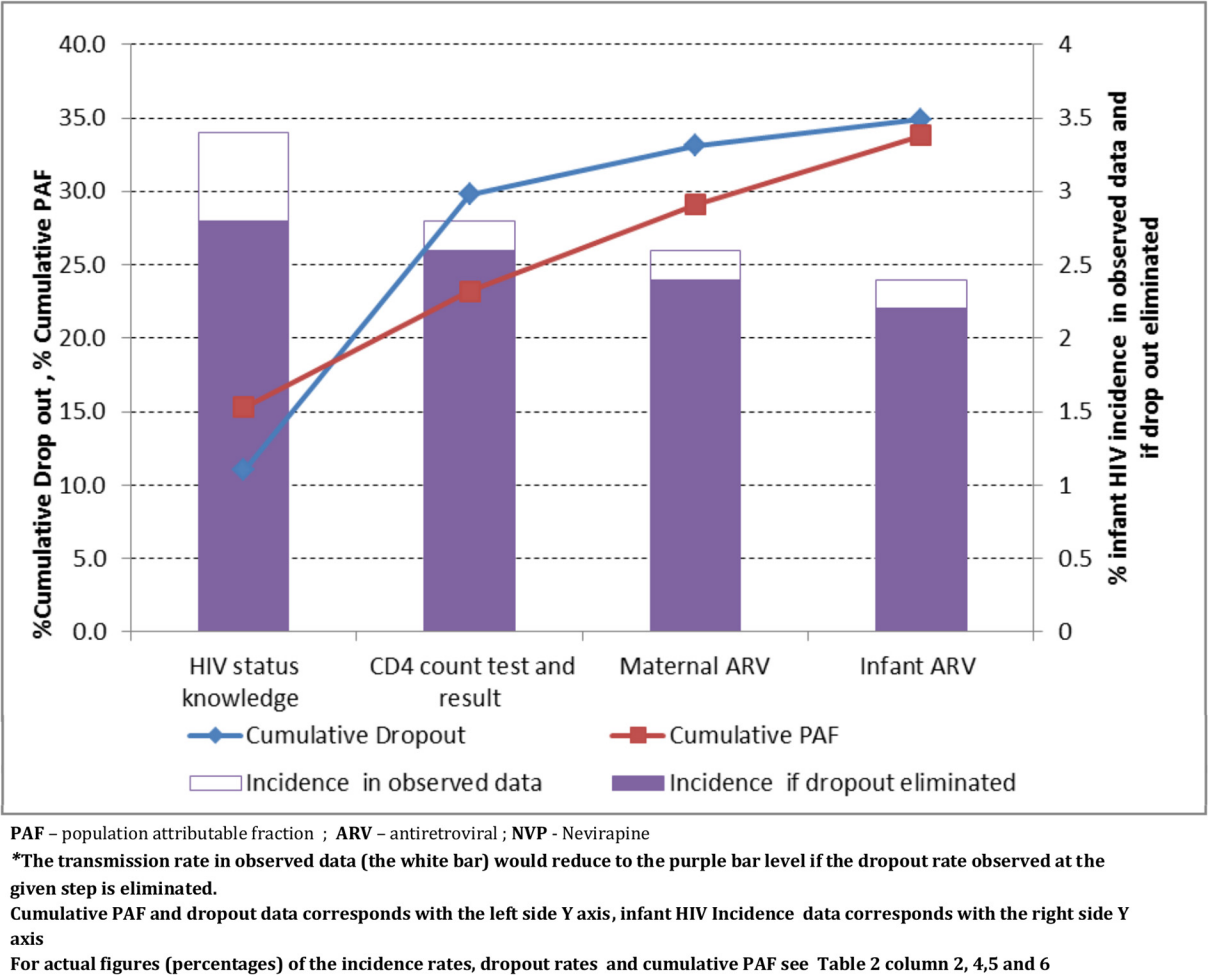
The increase in cumulative PAF as cumulative dropout rate increases (red and blue lines), and reduction in infant HIV incidence if dropout is eliminated (purple bar*).

In the cumulative PAF, overall we observed an increasing trend in PAF as cumulative dropout rates increased (Fig 2). The PAF of dropouts for all steps in the cascade combined is 33.8% (95% CI: 14.9, 48.8)– 6.5% of the cumulative PAF was attributable to the CD4 count step whilst the remaining 27.3% PAF was attributable to the HIV testing, maternal and infant ARV steps. The HIV transmission rate predicted for two scenarios indicate the HIV transmission could be reduced from 3.4% (95% CI: 2.8, 4.1) in the observed data scenario (i.e. with 34.9% overall dropout rate) to 2.2% (95% CI: 1.6, 3.1) in an ideal scenario where no dropout occurs in the four key PMTCT cascade steps. Transmission could be reduced from 3.4% in the observed data to 2.8%, 2.6%, and 2.4% if missed opportunity at HIV status knowledge, CD4 count test and maternal ARV points, respectively, is prevented. In the cumulative PAF of 33.8%, maternal HIV status knowledge contributes nearly half of this fraction and thus half of the reduction in transmission from 3.4% to 2.2% (Table 2 and Fig 2).

The specific PAF model fitted for estimating the contribution of mothers who did not receive maternal ARVs shows that 26.6% (95%CI: 13.0–38.1) of the transmission could have been prevented if all HIV-positive mothers in the study received some ARV during pregnancy (Table 2).

## Discussion

This national study shows high uptake of antenatal HIV testing (97.8%) and 80.4% uptake of both maternal and infant ARVs among HIV-exposed infants. Significant improvement has been made in increasing uptake of PMTCT service in South Africa. Since the national scale-up of the PMTCT programme in 2002 the uptake of antenatal testing has doubled.[21] A number of interventions, including offering universal HIV testing at antenatal clinics, use of rapid tests for diagnosis of HIV infection, and increased access to ARV treatments, have made a notable contribution to the success of the South African PMTCT programme.[22] Despite this success, South Africa is still one of the highest burden countries globally, accounting for 8% (21,000/260,000) of new infant infections in sub-Saharan countries in 2012.[5] By analysing the early PMTCT cascade from maternal HIV testing to infant ARVs at delivery, we identified several key gaps that contribute to the current infant HIV infection burden, which can be targeted for improvement.

Our analysis shows that 33.8% of infant HIV infections at the early (4–8 weeks) postnatal period were attributable to missed opportunities at one or more steps of the PMTCT cascade–this is a modifiable risk that could be averted by ensuring that all HIV-positive mothers receive timely testing, CD4 count test and result, and maternal and infant ARV regimens. The treatment guideline in place at the time of this study was the 2006 World Health Organization (WHO) guideline. The guideline has since been revised to include the use of more efficacious ARV regimens. The latest guideline (i.e. WHO Option B+) recommends that all HIV-positive mothers be initiated with lifelong ARV treatments regardless of CD4 count or clinical stage. This removal of the CD4 count requirement for initiating ARVs eliminates dropouts at CD4 count step and averts the transmission risk attributable to dropouts at this step. Nonetheless, in this study the majority (27.3% of the 33.8% PAF) of the transmission risk was attributable to the HIV testing and initiation of maternal and infant ARV steps; whilst 6.5% of the transmission was attributable to the CD4 count step [the small PAF attributable to CD4 count step could probably be because most mothers who dropped out at CD4 count step received ARVs regardless of CD4 count].Given the large attributable risk associated with missed opportunities at HIV testing and ARV steps, reducing missed opportunities at these steps should be prioritized in the current WHO Option B+ based PMTCT programme. Our findings show eliminating all dropouts (in other words achieving 100% coverage of each step of the PMTCT cascade) could potentially reduce the current 3.4% transmission rate, which resulted from an overall dropout rate of 34.9%, to 2.2%, almost enabling the achievement of the national PMTCT target for early transmission. Our study did not assess adherence to treatment hence the measured attributable risk and reduction of transmission rate is only for eliminating dropouts from the four service points.

Half (16.7%) of the total (33.8%) transmission risk attributable to the PMTCT cascade service was accounted for by unawareness of HIV status. HIV testing at first antenatal visit remains a crucial service for early detection of HIV in pregnant women regardless of the type of treatment option implemented. The coverage of antenatal HIV testing in this study was high (97.8%) and has enabled the detection of most (89.0%) HIV-positive mothers. However a significant percent (8.6%) of mothers who reported testing HIV-negative antenatally were found HIV-positive from our infant antibody test. The possible explanations for this discordant result have been discussed in detail in other papers.[23, 24] One of the main reasons given for the discordant result is possible new maternal infections/sero-conversion that occurs during pregnancy due to a general lack of HIV prevention counselling and couples testing services antenatally. Given women who test HIV-negative at first antenatal visit remain at risk of acquiring HIV during pregnancy,[23] targets for antenatal HIV testing should strive to achieve 100% coverage in first antenatal HIV testing, improved repeat antenatal and delivery HIV testing (to identify new infections), and high coverage for couples testing and counselling on safe sex.[24–26] The discordance in the reported maternal HIV status and the six-week HIV test result could also be due to maternal false report or unwillingness to disclose HIV-positive status.

The highest dropout rate (18.3%) was observed at the CD4 count testing step. Dropout at CD4 count testing is often associated with lack of onsite CD4 count testing service (e.g. long turnaround time). The introduction of WHO-recommended PMTCT treatment options of B (cART from early pregnancy through breastfeeding) and B+ (life-long cART) totally eliminates this problem by removing the CD4 testing prerequisite for cART initiation. However, treatment adherence and retention of mothers through the postnatal period (or for life-time in the case of option B+) are the other important concerns that need close monitoring as South Africa and other countries move to treatment options B and B+. The importance of access to ARVs is emphasised in this study. Our findings show more than a quarter (26.6%) of the mother-to-child transmissions are preventable by ensuring mothers receive some antenatal ARV.

This study identifies the following groups of mothers who were more likely to miss one or more services along the PMTCT cascade: adolescent (<20years) mothers, primipara mothers, those who had home deliveries, mothers presenting late (during 3^rd^ trimester) in their pregnancy for antenatal services, low socioeconomic group, low educational status, and non-disclosure of HIV status. Most of the identified risk factors were influential across the 4 cascade steps, showing their overall strong effect on uptake of ARVs. Addressing these risk factors should thus be a priority for current PMTCT programmes. We postulate community-level interventions, including community awareness raising, promoting women education and strengthening linkages and referral-system between communities (e.g. community health workers, mentor mothers) and health facilities would improve access to PMTCT services. Some of these interventions (community-based programmes/community health workers) are already proven to be effective for improving uptake of cART and retention along the PMTCT cascade.[27, 28]

We found adolescent (<20 years) mothers were both at high risk of poor PMTCT service uptake and had a significantly higher rate of unplanned pregnancies compared to adult (≥ 20 years) mothers. Being an adolescent was the strongest predictor of unawareness of HIV-positive status. The majority of mothers who were unaware of their HIV-positive status at delivery reported a negative HIV test during antenatal visits, suggesting that either these women were unwilling to report their positive status or that HIV infection among these mothers occurred around conception or thereafter. Interventions targeting sexually active adolescent girls are necessary in order to reduce both the risk of contracting HIV during conception (or thereafter) and unplanned pregnancies in the adolescent age group. Our findings on both the unplanned pregnancies and unawareness or denial of HIV status among adolescent mothers are consistent with other studies in South Africa.[10, 14]

Our study has several limitations. Although the study measured uptake of PMTCT services for the nation, children who had died or were ill by the age of 4–8 weeks, children who did not receive first immunisation at public facilities and children who received first immunisation after 8 weeks of age were not included in this study. However most immunisations occur in PHCs and attendance of six-week immunisation clinic in South Africa is very high (>95%), hence our results represent the majority of infants. The demographic profile and HIV prevalence (among mothers) in this study is very similar with the 2009 antenatal survey further validating the generalizability of our study estimates. Repeat antenatal testing, timing of antenatal testing, uptake of early infant diagnosis, cotrimoxazole coverage and uptake of postnatal PMTCT services were not captured in this study. We therefore recommend that these indicators be added in future studies. The data on all PMTCT cascade indicators were collected from interviews with mothers. Recall bias could be expected on certain PMTCT cascade indicators, but this is likely to be minimal as questions were restricted to basic information that were tied to specific clinic visits. Visual aids (such as pictures of ARV pills and ARV syrup bottles) were also used to assist mothers to identify the type of regimen received. Our estimate of unawareness of HIV-positive status could be an over estimate if mothers reported HIV-negative status due to fear of stigma or denial. We checked Road-to-health cards to verify maternal self-report of HIV status and ARV uptake, therefore we expect this will minimize information bias due to false-reports and recall bias. Lastly, the majority (66.2%) of infant infections were unaccounted for by gaps in the PMTCT cascade. Given that we did not control for factors such as maternal health status (CD4 count/viral load), adherence to treatment, and duration and type of regimens, this result is expected. Future studies should attempt to include these other factors to estimate the attributable risk of early peri-natal transmission.

Methods applied in this study to quantify the population attributable risk should be used more often in future studies so that informed decision can be made in prioritizing between alternative interventions/strategies for addressing barriers for PMTCT uptake.

## Conclusion

Our findings indicate that a third of infant HIV infections detectable at 6 weeks are attributable to missed opportunities of key PMTCT services and can be prevented by optimizing the uptake of existing key antenatal and early postnatal PMTCT services and this could potentially reduce HIV transmission to 2.2%. Risk factors for low PMTCT service uptake should be addressed through health facility and community-level interventions, including community awareness raising, promoting women education, strengthening linkages and referral-system between communities and health facilities, improving repeat antenatal and delivery testing services, and interventions targeting sexually active adolescent girls to prevent both maternal HIV infection during conception and unplanned pregnancies in this group.
